# Study protocol: a randomised controlled trial of a nurse-led community-based self-management programme for improving recovery among community-residing stroke survivors

**DOI:** 10.1186/s12913-016-1642-9

**Published:** 2016-08-15

**Authors:** Suzanne Hoi Shan Lo, Anne Marie Chang, Janita Pak Chun Chau

**Affiliations:** 1 School of Nursing, Faculty of Health, Queensland University of Technology, Brisbane, Queensland 4059 Australia; 2 The Nethersole School of Nursing, Faculty of Medicine, The Chinese University of Hong Kong, Shatin, N.T. Hong Kong

**Keywords:** Stroke, Disease management, Self-efficacy, Community-dwelling, Quality of life, Community reintegration, Randomised controlled trial

## Abstract

**Background:**

Recovery after stroke is long-term and demanding. Optimising community-residing stroke survivors’ capability to self-manage their health is integral. Recent systematic reviews have shown that stroke self-management programmes were associated with significant improvement in stroke survivors’ health-related quality of life and self-efficacy. However some programmes were not designed with an underpinning theoretical framework. The aim of this study is to compare the effectiveness of a nurse-led stroke self-management programme with usual care on recovery of community-residing stroke survivors.

**Methods/Design:**

A single-blinded, two-arm, randomised controlled trial will be conducted. Patients with a history of first or recurrent ischaemic or haemorrhagic stroke who will be discharged to home settings will be recruited from acute stroke units of three acute public hospitals in Hong Kong. The estimated sample size is 160 (80 participants per group). Eligible participants will be randomised to receive either usual care or a 4-week nurse-led community-based self-management programme plus usual care after discharge. The programme, underpinned by Bandura’s constructs of self-efficacy and outcome expectation, includes one individual home visit, two community-based group sessions, and three follow-up phone calls. Primary outcomes include stroke survivors’ self-efficacy and outcome expectation of performing self-management behaviours. Secondary outcomes include health-related quality of life, satisfaction with performance of self-management behaviours, depressive symptoms, and community reintegration. Participants will be assessed at baseline and at 8 weeks after randomisation. Generalised estimating equations will be performed to evaluate the significance of changes in outcomes over time by treatment condition. Research ethics approvals were obtained.

**Discussion:**

It is expected that stroke survivors receiving the stroke self-management programme will have improved self-efficacy, outcome expectation, and performance of stroke self-management behaviours. Enhanced quality of life and level of community reintegration, and decreased depressive symptoms are also expected. The study results will provide valuable evidence to inform future identification and evaluation of best approach to deliver stroke self-management programmes to enhance community-residing stroke survivors’ recovery.

**Trial registration:**

ClinicalTrials.gov identifier: NCT02112955; date of registration: 09/04/2014

## Background

Stroke is the second leading cause of global death with the death toll amounting to 6.7 million (11.9 % of all deaths) in 2012 [1]. In Hong Kong, stroke was the fourth leading cause of death in 2014 [2]. Stroke is a significant cause of disability with survivors having a higher risk of depressive symptoms and a lowered quality of life [3]. Indeed stroke recovery is long-term and demanding, encompassing not only physical rehabilitation, but also coping with cyclic frustrations and adapting to new life roles [4, 5]. Previous studies found poorer functional ability, more depressive symptoms, and lower self-esteem were significantly associated with participation restriction 12 months post-stroke [6]. The period for optimal recovery inevitably extended far beyond in-patient rehabilitation. The level of complexity in managing physical and psychosocial needs after stroke is intensified when stroke survivors return home and healthcare professionals are not immediately available to provide support. Furthermore when at home support from family may decrease as they return to their normal life [7]. Provision of effective community-based interventions to optimise stroke survivors’ capability to manage their post-stroke challenges is important to foster independence and early reintegration to pre-stroke life.

Self-management refers to an individual’s active participation in managing the symptoms, treatment, physical and psychosocial consequences of a chronic illness. There are three main components of chronic disease self-management including medical, emotional and role management [8]. To attain effective self-management, practicing core self-management skills including goal-setting, problem-solving, decision-making, resources utilisation and communication with healthcare professionals is crucial [4]. A recent systematic review of nine randomised and six non-randomised studies reported benefits of stroke self-management programmes in physical outcomes, quality of life, and confidence in recovery among stroke survivors. However the essential contents and the best approach to deliver the programmes for stroke survivors are yet to be determined [9].

Stroke self-management programmes are complex interventions given the interaction of multiple components of interventions with varying strengths, doses, or contents. According to the Medical Research Council guidance [10], designing complex interventions with an underlying theoretical premise enables its systematic development and implementation, and informs selection of appropriate outcome indicators. Furthermore it helps predict and explain the mechanism of change in target behaviours or the relationship between interventions and outcomes. A recent systematic review was conducted to examine the effectiveness of theory-based stroke self-management programmes [11]. The review included three randomised controlled trials examining three community-based programmes based on the Stanford Chronic Disease Self-management programme underpinned by Bandura’s construct of self-efficacy. The results showed potential benefits of the programmes in improving stroke survivors’ self-efficacy and health-related quality of life. However only two of the three included studies explicitly described how the theory informed programme design and implementation, and measured stroke survivors’ changes in self-efficacy. The other study neither linked the programme to Bandura’s four sources of self-efficacy nor measured self-efficacy [11]. There is also insufficient evidence on effectiveness of different strategies to enhance community-residing stroke survivors’ self-efficacy in managing their health conditions. All studies were conducted in Western countries and were delivered by healthcare professionals or trained peer leaders [11]. There has been no study which assessed the effectiveness of stroke self-management programmes delivered by nurses for Chinese stroke survivors.

In Hong Kong, stroke patients, after having their acute medical conditions stabilised, are either transferred to rehabilitation hospitals for continued rehabilitation, or discharged directly to their homes, old aged homes or other residential care homes depending on their progress. Community-based support for stroke survivors, particularly for those after returning home, often includes stroke support groups, private physiotherapy or occupational therapy, and social services offered by non-government organisations such as vocational training. However, support programmes for community-residing stroke survivors to promote their psychological and social well-being, as well as to enhance their knowledge and skills for better reintegration into the community and assumption of post-stroke social and life roles are relatively insufficient. Given the potential programme benefits, it would be worthwhile to determine the effectiveness of a community-based nurse-led stroke self-management programme underpinned by a theoretical framework for Chinese stroke survivors. The findings of such a study would inform future identification and evaluation of best strategies to deliver effective stroke self-management programmes.

### Research aim

To examine the effectiveness of a nurse-led community-based self-management programme for improving recovery among Chinese community-residing stroke survivors.

### Research objectives

To determine the effectiveness of the programme on improving community-residing stroke survivors’

- self-efficacy, outcome expectation, and satisfaction with performance of self-management behaviours;
- health-related quality of life;
- depressive symptoms; and
- community reintegration.

## Methods/Design

### Study design and setting

**Fig. 1:**
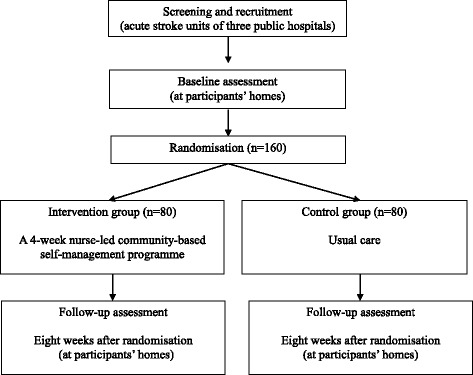
Study design and flow of participants

A two-arm, single-blinded randomised controlled will be conducted (Fig. 1). Participants will be recruited from acute stroke units of three public hospitals in Hong Kong. The nurse-led community-based self-management programme will be conducted at both the participants’ homes and a community centre after the participants have been discharged home from the hospital.

### Ethics consideration

Ethical approvals have been obtained from the Kowloon West Cluster Research Ethics Committee, Hospital Authority, Hong Kong (Ethical Approval No.: KW/EX-14-021(71–21)), the Joint Chinese University of Hong Kong-New Territories East Cluster Clinical Research Ethics Committee, Hong Kong (CREC Ref. No.: 2014.375-T), and the Human Research Ethics Committee of the Queensland University of Technology (Approval No.: 1400000333). Written informed consent will be collected from participants before commencing data collection. All potential participants will receive a full explanation by the research assistant about the purpose and data collection process of the study, their rights to voluntarily participate, refuse, or withdraw from the study without any negative consequences to them. The questionnaires will be anonymous and will be used for research purposes only. All the information collected will be kept strictly confidential and will be destroyed six years after completion of the study. The federal and institutional ethical standards, Hong Kong Personal Data (Privacy) Ordinance, Declaration of Helsinki, and ICH-GCP will be upheld.

### Participants

All stroke survivors admitted to the acute stroke units will be recruited if they 1) are aged 18 years or above; 2) have a clinical diagnosis of a first or recurrent ischaemic or haemorrhagic stroke (inclusive of intracerebral haemorrhage and subarachnoid haemorrhage); 3) will be discharged from hospital to home in the next seven days; 4) have a Mini Mental State Examination (MMSE) score >18 measured at the time of consenting to participate in the study [12]; 5) can speak Cantonese; and 6) can attend sessions of the self-management programme.

Participants will be excluded if they 1) are diagnosed with transient ischaemic attack, subdural or epidural haemorrhage [13]; 2) have cerebrovascular events due to malignancy or head trauma; 3) have limited comprehension and receptive aphasia; 4) have been diagnosed with schizophrenia, bipolar disorder, or dementia; or 5) have received a stroke self-management programme in the past 12 months.

### Sample size estimation

Primary outcome of this trial is stroke survivors’ self-efficacy in performing self-management behaviours measured by the Stroke Self-Efficacy Questionnaire at eight weeks after randomisation. A sample size of 64 per group (128 for two groups) is required to detect a significant mean difference in self-efficacy between groups for a medium effect size of 0.5 [14] with significance set at 0.05 and a power of 0.8 [15]. Taking into consideration an attrition rate of 19 % over six months among stroke survivors [14], the final sample size is 160 (80 participants per group). Recruitment of participants is anticipated to take 12 months.

### Randomisation

Eligible participants, immediately after providing baseline assessment data, will be randomly assigned in a 1:1 ratio to the intervention or the control group according to a computer-generated random schedule in permuted blocks of six with no stratification. A statistician who is not involved in recruitment, treatment allocation, and outcome assessment will generate the randomisation numbers. The statistician will put the randomly sequenced numbers indicating group assignment into sealed, opaque, containing a carbon paper inside, and sequentially numbered, otherwise identical, envelopes. Upon enrolling each participant, a research assistant who is not involved in recruitment and outcome assessment will take an envelope consecutively, write the participant’s study identifier on the envelope, open it and inform the principal investigator about the treatment allocation.

### Blinding

Due to the nature of the intervention, the participants and the principal investigator who delivers the self-management programme will not be blinded to treatment allocation. However the research assistants who will administer the outcome assessment, data entry and analysis will remain blinded to the treatment allocation. The research assistants will not be involved in implementing the programme or treatment of the participants at any stage. The participants will be asked not to tell the research assistants about their treatment allocation. However the research assistants who will collect the participants’ feedback on usefulness of the programme will not be blinded to treatment allocation as they need to know this information for conducting the assessment.

### Intervention

#### Intervention group: a nurse-led community-based self-management programme

Participants assigned to the intervention group will receive a 4-week nurse-led community-based self-management programme in addition to usual care. The programme is aimed at enhancing community-residing stroke survivors’ capabilities in self-managing their post-stroke health conditions. It includes one 1.5-hour individual home visit, two 1.5-hour group sessions held in a community centre, and three follow-up phone-calls (Table 1). The programme is underpinned by Bandura’s constructs of self-efficacy and outcome expectation [16]. The programme will be delivered by the principal investigator who is a registered nurse experienced in stroke care and chronic disease management. Each participant will receive a programme booklet and two DVDs specifically designed for this programme. The booklet provides information about stroke recovery and self-management, and records participants’ goals and action plans. The DVDs contain video clips of experience sharing by 15 stroke survivors who have successfully managed their stroke. A panel of experts including two nurse academics, two advanced practice nurses, one nurse manager and one physician who are experienced in stroke care has reviewed the programme contents and the booklet. A protocol detailing the programme contents and delivery methods have been developed to ensure consistent delivery of the programme. The programme will commence when six participants have been assigned to the intervention group and repeated to accommodate all participants assigned to this band. Family members or informal carers of each participant who are interested may join the programme as an accompanying person.

**Table 1:** Overview of the nurse-led community-based self-management programme for community-residing stroke survivors

Week	Programme components	Key contents
1	Home visit (at participant’s home)	- Perform an individualised assessment. - Provide information about stroke self-management. - Establish a short-term goal and an action plan. - Video viewing.
2	Group sessions (in a community centre)	- Discuss the physical and psychosocial consequences of stroke. - Discuss the practical tips of managing post-stroke challenges. - Facilitate reflection and experience sharing among the group. - Explore alternative ways to better implement the action plans. - Practice the use of core self-management skills. - Video viewing.
3 & 4	Three follow-up phone calls	- Review progress towards goal attainment. - Provide individualised feedback and positive reinforcement. - Encourage to continue, revise, or set a new short-term goal.

### Theoretical framework of the self-management programme

**Fig. 2:**
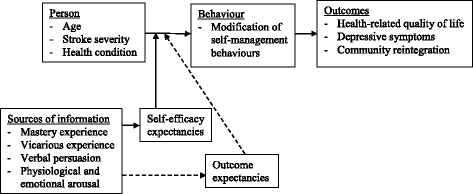
Theoretical framework of the stroke self-management programme. (Adapted from Bandura [16])

Bandura’s construct of self-efficacy [16] will be adopted to guide the proposed programme design and implementation. Self-efficacy refers to an individual’s confidence to perform an action to reach a desired goal [16]. Judgment of one’s self-efficacy determines the course of action required, degree of effort, and perseverance to continue an action even in face of obstacles. Self-efficacy is developed through four sources of information namely mastery experience, vicarious experience, verbal persuasion, and minimising physiological or emotional arousal [16]. Substantial literature reported increased self-efficacy leads to modification of health behaviours, and better patient outcomes such as quality of life or perceived health status [9, 11]. Outcome expectation, another central construct of Bandura’s social cognitive theory, refers to an individual’s judgement of the likelihood that performance of a particular action will produce a certain outcome. It takes three major forms including physical, social and self-evaluative effects. Within each form, positive expectations serve as incentives while negative expectations serve as disincentives to one’s performance of an activity [16]. Bandura suggests that the initiation and continuation of a particular activity or behaviour would be best predicted by the combined influence of self-efficacy beliefs and types of outcome expectation. The stronger one believes in own ability to perform a specific activity and the outcome of that activity, the more likely that one will initiate and maintain the behaviour [16, 17]. In this study, the influences of self-efficacy and outcome expectancy beliefs on stroke survivors’ performance in stroke self-management behaviours will be addressed when designing the programme. Figure 2 outlines the theoretical framework of the programme. Table 2 summarises the strategies adopted to enhance participants’ self-efficacy and outcome expectation of performing self-management behaviours.

**Table 2:** Strategies adopted to enhance participants’ self-efficacy and outcome expectation of performing self-management behaviours

Sources of information of self-efficacy	Strategies adopted	Programme components
Mastery experience	- Establish a short-term goal and a realistic action plan. - Assist to break down difficult tasks into simple steps. - Practice the use of core self-management skills. - Revise the action plan if necessary. - Encourage to record the implementation of the action plan.	HV HV, GS GS GS, PF HV, GS, PF
Vicarious experience	- Guide the viewing of videos about experience sharing. - Facilitate experience sharing among the group.	HV, GS GS
Verbal persuasion	- Acknowledge incremental successes. - Provide positive reinforcement. - Reinforce the importance of ‘taking an active role’.	HV, GS, PF HV, GS, PF HV, GS, PF
Physiological & emotional arousal	- Assist to reinterpret negative physiological and emotional states.	HV, GS, PF
Outcome expectation	- Assess and reinforce the positive outcomes valued by the participants after performing stroke self-management behaviours.	HV, GS, PF

*GS* Group sessions, *HV* Home visit, *PF* Follow-up phone calls

### Programme contents

The nurse will schedule with the participants for home visit. During home visit, the nurse will assess the participant’s current medical, emotional and role management of their post-stroke conditions [4, 8], and their self-efficacy and outcome expectancy beliefs of performing self-management behaviours. Based on the assessment, the nurse will work with the participant to establish a short-term goal of importance to stroke recovery, and develop a realistic action plan to achieve the goal [4]. Information about stroke self-management and core self-management skills such as problem-solving and decision-making will be provided. Furthermore participants will be guided to view one to two video clips on the DVDs to enable them to learn from other stroke survivors’ experiences. Positive reinforcement will be provided to promote their confidence in implementing their action plans [16]. Participants will be encouraged to view the videos, read the programme booklet, and record the implementation of their action plans after the home visit.

In the following week after the home visit, two group sessions will be held on the same day in a community centre. During the group sessions, participants will be provided with information about the physical and psychosocial consequences of stroke, and practical tips for effective stroke self-management. More importantly, the nurse will act as a facilitator to encourage participants to reflect and share their barriers to and facilitators of implementing their action plans. Based on the common problems among the group, video clips of sharing by stroke survivors will be presented. Participants will be facilitated to explore own ways to address their barriers. Examples will be provided to guide their practice of core self-management skills to better implement their plans. If necessary, their action plans will be revised. Positive reinforcement will be provided and participants are encouraged to visualise their expected positive outcomes after performing the self-management behaviours [16]. Participants will be encouraged to continue implement their action plans and record their progress after the sessions.

Three weekly follow-up phone calls by the nurse will be provided after the group sessions to review the participant’s progress, barriers to and facilitators of implementing the action plans. Individualised feedback and positive reinforcement will be provided. Participants’ expected positive outcomes of achieving the goals will be reinforced. At the end of the last follow-up phone call, participants’ extent of goal attainment will be evaluated. They will be guided to continue, revise, or establish a new short-term goal for the next month.

### Control group: usual care

Participants in the control group will receive usual care provided to stroke survivors discharged to their home without any additional interventions. Usual care includes some or all of the following services: hospital- or community-based health education for example talks or leaflets; follow-up appointments in an outpatient clinic or a day hospital; medical consultations with a local general practitioner; referral to or information about local community-based services for people with stroke or disability for example meals on wheels.

### Outcome measures

#### Primary outcomes

##### Self-efficacy

The 13-item Stroke Self-Efficacy Questionnaire (SSEQ) will be used to measure participants’ self-efficacy in performing daily functional activities and self-management such as getting in or out of bed, preparing meals, or persevering to make progress [18]. Each item is rated on an 11-point Likert scale (0 = ‘not at all confident’; 10 = ‘very confident’). The score of all items is summed to yield the total score (range 0–130). A higher score represents higher self-efficacy in performing daily functional activities and self-management. The authors have back-translated [19] and validated the Chinese version of SSEQ in a convenience sample of 135 Chinese community-residing stroke survivors. The results showed high internal consistency (Cronbach’s alpha = 0.92). There were positive correlations between the total scores of the translated Chinese version of SSEQ, and General Self-Efficacy Scale (Spearman’s rho *r*_*s*_ = 0.48, *p* < 0.01) and Frenchay Activities Index (Spearman’s rho *r*_*s*_ = 0.51, *p* < 0.01), suggesting acceptable convergent validity.

##### Outcome expectation

An 11-item Chinese version of the Stroke Self-management Outcome Expectation Scale was developed based on Bandura’s construct of self-efficacy [16], and previous literatures on stroke self-management [4] and outcome expectation [20, 21] to measure participants’ current level of confidence that the desired outcomes will be resulted after performing the self-management behaviours. Each item is rated on an 11-point Likert scale (0 = ‘not confident at all’; 10 = ‘very confident’). The total score is summed (range 0–110). A higher score indicates higher confidence in likelihood of the outcomes to occur. An expert panel of two nurse academics, one nurse manager, three advanced practice nurses and two physicians performed assessment of content validity (Content validity index: 0.98). The scale was piloted in a convenience sample of 83 Chinese community-residing stroke survivors and showed high internal consistency (Cronbach’s alpha = 0.94).

#### Secondary outcomes

##### Stroke self-management behaviours

An 11-item Chinese version of the Stroke Self-management Behaviours Performance Scale was developed by the authors based on previous literatures about stroke recovery to measure participants’ satisfaction with their current performance in stroke self-management behaviours [4, 22, 23]. Each item is rated on an 11-point Likert scale (0 = ‘very dissatisfied’; 10 = ‘very satisfied). The total score is obtained by summing the scores of all items (range 0–110). A higher score indicates higher level of satisfaction with own performance in self-management behaviours. An expert panel of two nurse academics, one nurse manager, three advanced practice nurses and two physicians performed assessment of content validity (Content validity index: 0.98). The scale was piloted in a convenience sample of 83 Chinese community-residing stroke survivors and showed high internal consistency (Cronbach’s alpha = 0.93).

##### Health-related quality of life

The 49-item Stroke Specific Quality of Life Scale (SSQOL) will be used to measure participants’ health-related quality of life [24, 25]. It consists of 12 domains including self-care, vision, language, mobility, work/productivity, upper extremity function, critical-thinking, personality, mood, family roles, social roles, and energy. Each item is rated on a 5-point scale (0 = ‘couldn’t do it at all’ to 5 = ‘no trouble at all’; or 0 = ‘strongly agree’ to 5 = ‘strongly disagree’). The scores are summed to yield the total score (range 49–245) and domain scores. A higher total score indicates better health-related quality of life [24]. The authors have back-translated [19] and validated the Chinese version of SSQOL in a convenience sample of 135 Chinese community-residing stroke survivors. The results showed high internal consistency (Cronbach’s alpha of the total and domain scores: 0.63–0.93). The total score of the translated Chinese version of SSQOL was significantly correlated with the Chinese version of SSEQ (Spearman’s rho *r*_*s*_ = 0.68, *p* < 0.01) and Frenchay Activities Index (Spearman’s rho *r*_*s*_ = 0.60, *p* < 0.01), suggesting acceptable convergent validity. An additional 13 items will be used to measure participants’ changes in each domain and the overall health-related quality of life compared with their pre-stroke condition. Each item is rated on a 4-point scale from 1 ‘same as’ to 4 ‘a lot worse’.

### Depressive symptoms

The 15-item Chinese version of the Geriatric Depression Scale will be used to measure participants’ presence of depressive symptoms in the past week [26, 27]. Each item is rated on a yes/no format. A score will be given to the ‘yes’ items. The total score is calculated by summing each item score (range 0–15). A score of nine or above suggests moderate or severe depression. The scale has high internal consistency (Cronbach’s alpha = 0.78) and acceptable convergent validity. Significant correlation was found between the total scores of the scale and the London Handicap Scale (*r* = −0.30, *p* < 0.01) [28].

### Community reintegration

The 11-item Chinese version of the Reintegration to Normal Living Index will be used to measure participants’ level of community reintegration after stroke [29, 30]. Participants will be asked about the extent to which each statement with regards to six domains (mobility, self-care, activities, role within the family, comfort with relationships, and ability to handle life events) describes their current situation. All items are rated on a 4-point scale (1 = ‘does not describe my situation’ to 4 = ‘fully describes my situation’). Two subscale scores ‘daily functioning’ and ‘perception of self’, and a total score will be summed and normalised to 100 (range 25–100). A higher score indicates higher perceived community participation. The index has high internal consistency (Cronbach’s alpha = 0.92) and good convergent validity. Significant associations were reported between the Index and Frenchay Activities Index (*r* = 0.44, *p* < 0.001), and Personal Wellbeing Index (*r* = 0.25, *p* = 0.033) [30].

### Usefulness of the programme

Participants receiving the self-management programme will be assessed about their: 1) extent of overall programme participation by calculating the average of participants’ percentage of attendance at the programme; 2) self-reported frequency of viewing the videos on the DVDs; 3) number of personal goals attained upon programme completion; 4) reasons for not being able to attain the goals; and 5) qualitative comments about usefulness of the DVDs and areas for further improvement of the programme.

### Demographic and clinical information

Demographic data of the participants including age, gender, educational level, occupation, marital status, living condition, accommodation, main carer, social history, and financial assistance will be recorded. Clinical information including duration after stroke onset, type and location of stroke, past health history, current medication regimen, medical follow-up, smoking habits, use of alcohol, use of assistive devices and health services utilisation will be recorded. Furthermore, physical examination will be performed by the research assistant using instruments including MMSE, Glasgow Coma Scale, National Institutes of Health Stroke Scale (NIHSS), and the Barthel Activities of Daily Living (ADL) Index.

### Data collection procedures

All potential participants will be identified by reviewing their medical records or referrals by stroke nurses at the study venues. The principal investigator or research assistants will screen the participants in a face-to-face interview for their eligibility and willingness to participate in the study. Written informed consent will be obtained from those who are eligible. A card indicating participants’ involvement in the study and means of urgent contact will be provided to each participant after obtaining the consent. The consented participants’ demographic and clinical information will be retrieved from their medical records. Baseline and follow-up assessments will be conducted by two research assistants. The participants will be scheduled via phone by a research assistant for baseline assessment within one month after their discharge from hospital. Immediately after baseline assessment is completed, the participants will be randomly assigned to either the intervention or the control group. At 8 weeks after randomisation, the participants will be scheduled via phone by the research assistant again for follow-up assessment. The research assistants will administer the assessment tools in a face-to-face interview at the participants’ homes. The administration of the questionnaires has been pilot tested among six Chinese community-dwelling stroke survivors (mean age 55 years, SD 10.35, range 35–64 years). It took an average of one hour to complete the six questionnaires. Another research assistant will call the participants in the intervention and control groups at the time immediately after programme completion to collect their feedback on the programme (Table 3). The research assistants will be trained to administer the assessment tools consistently and non-judgmentally; and be reminded of the importance of confidentiality and safe storage of data. Inter-rater reliability between research assistants will be checked using Kappa statistics before data collection.

**Table 3:** Summary of measurements and study outcomes

Measurements	Instruments	Group	Baseline	Follow-up	Outcomes	
					1°	2°
Self-efficacy	SSEQ	IG, UG	×	×	×	
Outcome expectation	SSOES	IG, UG	×	×	×	
Satisfaction with performance of SSMB	SSBPS	IG, UG	×	×		×
Health-related quality of life	SSQOL	IG, UG	×	×		×
Depressive symptoms	GDS	IG, UG	×	×		×
Community reintegration	RNLI	IG, UG	×	×		×

*GDS* Geriatric Depression Scale, *IG* Intervention group, *RNLI* Reintegration to Normal Living Index, *SSBPS* Stroke Self-management Behaviours Performance Scale, *SSEQ* Stroke Self-Efficacy Questionnaire, *SSMB* Stroke self-management behaviours, *SSOES* Stroke Self-management Outcome Expectation Scale, *SSQOL* Stroke Specific Quality of Life Scale, *UG* Usual care group

1°: Primary outcome

2°: Secondary outcome

### Non-participant observation

An expert panel member will conduct non-participant observation of the first session of the programme. The purpose is to identify the extent to which the programme was delivered according to the protocol using a Likert scale from 1 ‘A small extent’ to 5 ‘A large extent’, participants’ level of participation, and facilitators of and barriers to their participation.

### Statistical analysis

Statistical analyses will be performed using the IBM SPSS Statistics version 22 (SPSS Inc., Chicago, IL, USA). All primary analyses will be conducted on an intention-to-treat basis. No interim analyses will be undertaken. Descriptive statistics will be used to summarise participants’ baseline characteristics and outcome variables. All continuous outcomes will be assessed for normality in distribution. Appropriate transformation of skewed data will be performed before analysis. Chi-square tests and independent t-tests will be used to compare the demographic and clinical characteristics, and mean scores of outcomes between the intervention and control groups at baseline. A generalised estimating equation (GEE) model will be employed to assess differential change of the primary and secondary outcomes across the time points (baseline and eight weeks after randomisation) between the two groups. Adjustment will be made for potential confounding demographic and clinical variables, including gender, age, number of strokes, NIHSS, and the Barthel ADL Index, that were ‘unbalanced’ between the two groups (*p* < 0.25) [31, 32]. The longitudinal association of self-efficacy and outcome expectation with secondary outcomes will be examined using GEE model. GEE model can account for intra-correlated repeated measures data and accommodate missing data caused by dropouts, provided the data are missing at random [33], and is particularly suitable for intention-to-treat analysis without imputation for missing data. Significance level will be set at two-sided *p* < 0.05 and all statistical tests will be two-tailed.

## Discussion

To the best of the authors’ knowledge, this is the first randomised controlled trial which examines the effectiveness of a nurse-led stroke self-management programme on recovery among Chinese community-residing stroke survivors. This study adopts Bandura’s constructs of self-efficacy and outcome expectation [16] which considers both efficacy and outcome expectancy beliefs and the methods for improving these, as the underlying theoretical premise to guide the programme design, implementation and evaluation. Maintaining hopes for positive outcomes would be important incentives to stroke survivors’ pursuance in stroke self-management behaviours given a certain level of physical functioning [4]. It is expected that stroke survivors receiving the stroke self-management programme will have improved self-efficacy, outcome expectation, and performance of self-management behaviours. Enhanced health-related quality of life and level of community reintegration, and decreased depressive symptoms are also be expected. The study results will provide valuable evidence to inform future identification and evaluation of best approaches to deliver stroke self-management programmes to enhance community-residing stroke survivors’ recovery.

## Abbreviations

ADL: Activities of Daily Living
GEE: Generalised Estimating Equation
MMSE: Mini Mental State Examination
NIHSS: National Institutes of Health Stroke Scale
SSEQ: Stroke Self-Efficacy Questionnaire
SSQOL: Stroke Specific Quality of Life Scale
